# Reducing mortality in severe sepsis with the implementation of a core 6-hour bundle: results from the Portuguese community-acquired sepsis study (SACiUCI study)

**DOI:** 10.1186/cc9008

**Published:** 2010-05-10

**Authors:** Teresa Cardoso, António Henriques Carneiro, Orquídea Ribeiro, Armando Teixeira-Pinto, Altamiro Costa-Pereira

**Affiliations:** 1 Unidade de Cuidados Intensivos Polivalente - Hospital Geral de Santo António, University of Porto, Largo Prof. Abel Salazar, 4099-001 Porto, Portugal; 2 Department of Biostatistics and Medical Informatics, CINTESIS, Faculty of Medicine, University of Porto, Alameda Prof. Hernâni Monteiro, 4200-319 Porto, Portugal

## Abstract

**Introduction:**

To evaluate the impact of compliance with a core version of the Surviving Sepsis Campaign 6-hour bundle on 28 days mortality.

**Methods:**

Cohort, multi-centre, prospective study on community-acquired sepsis (CAS).

**Results:**

Seventeen intensive care units (ICU) entered the study. Over a one year period, 4,142 patients were enrolled in the study. Of the 897 (24%) admitted with CAS, 778 (87%) had severe sepsis or septic shock on ICU admission. In the first six hours of hospital admission: (1) 62% had serum lactate measured; (2) 69% fluids administered; (3) 77% specimens collected for microbiology before antibiotic administration; (4) 48% blood cultures obtained; (5) 52% antibiotics administered within the first hour of the diagnosis; (6) vasopressors were given in 78%; (7) 56% had central venous measurement (CVP) measurement; (8) 17% had a central venous oxygen saturation (ScvO2) measurement; (9) dobutamine was administered in 52%. Compliance with all actions 1 to 6 (core bundle) was associated with an odds ratio (OR) of 0.44 [95% confidence interval (CI) = 0.24-0.80] in severe sepsis and 0.49 (95% CI = 0.25-0.95) in septic shock, for 28 days mortality. This corresponded to a number needed to treat of 6 patients to save one life.

**Conclusions:**

Compliance with this core bundle was associated with a significant reduction in the 28 days mortality. Urgent action should be taken in order to ensure that early sepsis diagnosis is followed by full completion of this "core bundle" followed by activation of expertise help in severe sepsis.

## Introduction

Despite great advances in our understanding of its pathophysiology, sepsis remains a major reason for hospital and ICU admission [1,2], associated with high morbidity, hospital resource use and mortality.

The escalating prevalence of severe sepsis and septic shock, combined with the devastating mortality, inspired the creation of an international effort to address the global consequences. The main goals of the Surviving Sepsis Campaign (SSC) are to increase awareness of sepsis among clinicians and the public, to develop guidelines for the management of severe sepsis and to foster a change in the management of septic patients with the aim of obtaining a 25% reduction in mortality over 5 years [3-5].

The implementation process of the SSC guidelines has gone through a process of 'bundle' definition. A bundle is a group of interventions related to a disease process, that when executed together, produce better outcomes than when implemented individually [6].

The six-hour bundle, called the resuscitation bundle, focuses on early identification, early goal-directed therapy and early antibiotics and cultures. These interventions should be available to all doctors working with severely ill patients and should be widely disseminated.

The 24-hour bundle includes administration of drotrecogin alfa per hospital guidelines, steroids in refractory septic shock, intensive glucose control and lung protective ventilation strategies. Aside from the fact that these were mainly reserved for use by intensive care physicians the clinical impact of the first three interventions is still controversial [7-9].

Along with the development of this campaign, a Portuguese network of ICUs was created in 2004. Designated as the study group, the network enrolled a large number of units from the north to south of Portugal representing 41% of all ICU beds. This is the largest and most detailed study on community-acquired sepsis (CAS) ever performed in Portugal. The SACiUCI study group objectives are to evaluate the epidemiology of CAS in patients who are admitted in Portuguese ICUs, to assess the level of compliance with the SSC guidelines recommendations and help improve adherence to these recommendations.

Part of the data of the SACiUCI study, regarding the influence of vasopressor agent in septic shock mortality [10] has already been published. The present analysis was performed to describe compliance with the SSC six-hour bundle and its impact in severe sepsis mortality.

## Materials and methods

### Study design

The SACiUCI study was a prospective, cohort, multi-centred study, conducted over one year (1 December, 2004 to 30 November, 2005) in 17 Portuguese ICUs. The ICU participation was by direct invitation/acceptance with no financial reward.

National and Hospital Research and Ethics Committee approved the study design and informed consent was waived due to its observational nature without any deviation from the current medical practice.

All adult patients (age ≥ 18 years) consecutively admitted in the participating ICUs were enrolled and screened for CAS. Patients were then followed up until death or hospital discharge.

### Definitions

Infection was defined as a pathologic process caused by the invasion of normal sterile tissue, fluid or body cavity by a pathogenic or potentially pathogenic microorganism (not believed to be a contaminant) and/or clinically suspected infection plus the prescription of antimicrobial therapy [11]. Community-acquired infection was defined as the onset of infection before hospital admission or not present at admission becoming evident in the first 48 hours [12]. Sepsis and sepsis-related conditions were defined according to the criteria proposed by the American College of Chest Physicians/Society of Critical Care Medicine [13]. For the analysis of compliance with the SSC bundles, only patients with severe sepsis on ICU admission were included, because time zero was defined as hospital arrival time.

The presence of underlying disease was recorded. Metastatic cancer, haematological malignancy and AIDS using Simplified Acute Physiological Score (SAPS) II definitions [14]; cirrhosis, chronic heart failure, chronic pulmonary failure using Acute Physiology and Chronic Health Evaluation II definitions [15]. Chronic renal failure if there was need of chronic renal support or history of chronic renal insufficiency with a serum creatinine level over 2 mg/dl); HIV status (without complications defining AIDS); haematological disease including chronic neutropenia (≥ 3 months) or ≤ 1000 PN/dL; immuno-compromised state was defined by either administration in the 12 months prior to ICU admission of chemotherapy, radiation therapy or the equivalent to 0.2 mg/Kg/day prednisolone for at least three months or 1 mg/Kg/day for a week within in the three months prior to ICU admission.

### Data collection and management

Data were collected prospectively using pre-printed case report forms, using a specific database software, or on line through the study web page. Training on data collection, including clarification of the SSC recommendations and bundles, was organised in three regional educational sessions: north, centre and south Portugal; all the responsible investigators were invited.

All data were collected using a web-based application developed by the Department of Biostatistics and Medical Informatics (Serviço de Biostatística e Informática Médica), Medical School, Unversity of Porto. Detailed instructions concerning the aims of the study and data collection were given to all participating centres and were also available at the study website [16] before starting data collection and throughout the study period. A medical doctor was individually designed as being responsible for data collection in each ICU. Periodically each ICU received a report with the errors and inconsistencies in the database and was requested to review them. The software program was also designed to identify and reject inconsistencies. The steering committee was easily accessible to all participating investigators by phone or e-mail to answer queries during the study.

Each case report form included 237 items. Data collection included demographic data and comorbid diseases. The SAPS II score in the first ICU day [14] and the Sequential Organ Failure Assessment (SOFA) score [17] during the first five days of ICU stay were also recorded. Microbiological and clinical infections data were reported, along with the antibiotics prescribed, their changes in prescription and duration of therapy.

The study was designed prior to the publication of SSC guideline bundle definition so a slightly different version of the bundles is studied. The six-hour bundle for severe septic patients consisted in having within the first six hours after hospital admission: 1) serum lactate measurement; 2) 500 to 1000 ml of crystalloids or 300 to 500 ml of colloids given over 30 minutes, and repeated as needed; 3) Other specimens (besides blood) obtained for microbiology before antibiotherapy is started; 4) blood cultures done; 5) antibiotic therapy administered within the first hour of the diagnosis; 6) vasopressors administered during and after fluid administration if mean arterial pressure (MAP) was less than 65 mmHg.

For patients with septic shock, in the same time frame, three additional interventions were considered: 1) central venous pressure (CVP) measurement as part of sepsis treatment/monitoring; 2) central venous oxygen saturation (ScvO2) measurement as part of sepsis treatment/monitoring; 3) dobutamine administered after fluids and vasopressors, if there were signs of low cardiac output, depending on clinical assessment.

The major differences for the SSC guideline bundle definition are: achievement of the target CVP of 12 cmH20 and ScvO2 of 70% or more (in this study the registering of specific values measured was not requested) and guide dobutamine infusion through the ScvO2 measurement (the need for inotropic infusion was left to the clinician's best judgement).

A 'yes' score for each action was obtained if it was executed in the pre-defined time frame and a 'no' score was obtained otherwise. Bundle compliance was computed as the proportion of actions completed for each patient.

### Statistical analysis

Descriptive analyses were made of the background variables. Pearson chi-square tests were used for categorical variables. T-tests were used to compare age and SAPS II score between groups. The Levene's test was computed to check the assumption of equal variances across groups. The variable length of hospital stay was highly skewed and a Mann-Whitney U test was used to compare differences between groups.

The core bundle compliance was divided into three categories: I) no completion (0 to 2 actions completed); II) partially completed (3 to 5 actions completed) and III) fully completed (all actions completed). If a patient was not eligible for one particular action, he or she could still be counted as having all actions completed as long as the remaining actions were completed.

Multiple logistic regressions were used to compute the odds ratio (OR) for each action and for the bundle compliance adjusted for type of sepsis (severe sepsis or septic shock), SAPS II, presence of comorbidities (any present or none), type of hospital (community vs university) and type of ICU (medical vs mixed). The covariates gender and source of sepsis were also considered for the logistic regression models but were not statistically significant and therefore not included in the models. Goodness of fit for all regressions was checked using Hosmer and Lemeshow test. All the tests accepted the goodness of fit. Statistical significance was defined as *P *< 0.05. The statistical analysis was performed in SPSS^®^16 (SPSS Inc., Chicago, IL, USA).

The number needed to treat (NNT) was computed using the OR for all actions completed and the predicted probability of death for patients with 0 to 2 actions of the six-hour bundle completed (PD_0-2_), through the formula [18].

## Results

**Figure 1:**
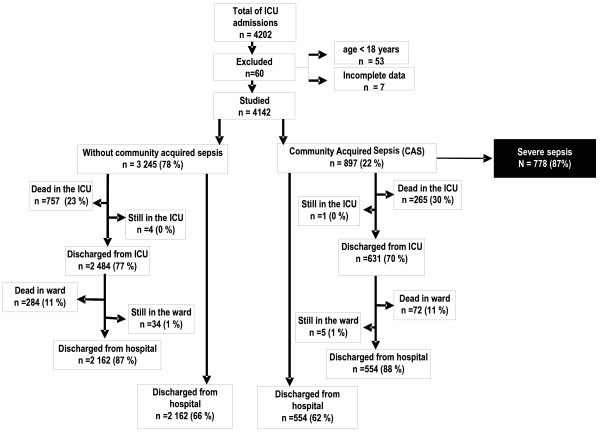
**Flow diagram of enrolled patients**.

Seventeen units entered the study from the north to south of Portugal corresponding to 41% of all national ICU beds, according to the 2001 Registry of the National Health Service (150 among 362 beds; Table 1). One unit, from a hospital with no emergency department, was excluded from further analysis because none of the septic patients admitted over the study period was considered to have CAS, increasing the mean incidence of CAS in the remaining 16 units to 24% (897 patients in a total of 3811; Figure 1).

**Table 1:** General characterisation of the total number of admissions in the participating units

	Type of hospital	Type of ICU	Number of beds	Total of ICU admissions n (%)	ICU LOS (median; IQR)		SAPS II (median; IQR)		CAS patients n (%)
Unit 1	Community	Medical	5	203 (5)	5	(2 - 9)	44	(33 - 60)	54 (27)
Unit 2	University	Mixed	6	177 (4)	6	(3 - 14)	38	(29 - 53)	22 (12)
Unit 3	Community	Mixed	6	253 (6)	4	(2 - 9)	37	(27 - 49)	76 (30)
Unit 4	Community	Medical	6	237 (6)	6	(4 - 11)	43	(32 - 56)	52 (22)
Unit 5	University	Mixed	5	124 (3)	5	(2 - 10)	45	(33 - 57)	22 (18)
Unit 6	Community	Mixed	8	291 (7)	6	(3 - 12)	43	(34 - 53)	63 (22)
Unit 7	University	Mixed	12	347 (8)	6	(3 - 12)	43	(32 - 52)	149 (43)
Unit 8	University	Mixed	12	300 (7)	10	(4 - 18)	50	(39 - 63)	28 (9)
Unit 9	Community	Mixed	11	331 (8)	3	(2 - 5)	37	(31 - 50)	0 (0)
Unit 10	University	Mixed	20	494 (12)	10	(6 - 19)	43	(33 - 53)	106 (21)
Unit 11	Community	Mixed	8	255 (6)	7	(4 - 13)	33	(24 - 43)	45 (18)
Unit 12	University	Medical	8	272 (7)	6	(3 - 13)	41	(28 - 56)	79 (29)
Unit 13	Community	Mixed	4	127 (3)	5	(3 - 11)	49	(33 - 58)	29 (23)
Unit 14	University	Mixed	11	366 (9)	6	(4 - 12)	45	(34 - 60)	96 (26)
Unit 15	University	Medical	8	166 (4)	7	(3 - 12)	48	(36 - 57)	41 (25)
Unit 16	Community	Mixed	6	82 (2)	3	(2 - 9)	26	(18 - 40)	8 (10)
Unit 17	University	Medical	14	117 (3)	10	(3 - 24)	53	(39 - 67)	27 (23)
**TOTAL**		**17**	**150**	**4142 (100)**	**6**	**(3 - 13)**	**42**	**(31 - 55)**	**897 (22)**

CAS: community-acquired sepsis; LOS: length of stay; IQR: interquartile range; SAPS: Simplified Acute Physiological Score.

General characteristics of the patients included in the study are shown in Table 2.

**Table 2:** General patient information

			Severity of sepsis					28-days outcome				
	Total(n = 778)		Severe sepsis (n = 341)		Septic shock (n = 437)		*P *value	Death (n = 257)		Alive (n = 521)		*P *value
**Age**, mean (SD)	61	(17)	59	(18)	63	(16)	**0.009**^#^	64	(16)	60	(17)	**<0.001**^#^
**Gender**, n (%)												
Female	280	(36)	129	(38)	151	(35)	0.345*	80	(31)	200	(38)	0.047*
Male	498	(64)	212	(62)	286	(65)		177	(69)	321	(62)	
**SAPS II**, mean (SD)	51	(19)	44	(15)	57	(19)	**<0.001**^#^	62	(21)	46	(16)	**<0.001**^#^
**Diagnosis on admission**, n (%)												
Medical	634	(81)	289	(85)	345	(79)	-	214	(83)	420	(81)	-
Trauma	6	(1)	3	(1)	3	(1)		0	(0)	6	(1)	
Surgical	138	(18)	49	(14)	89	(20)		43	(17)	95	(18)	
**Focus of infection**, n (%)												
Respiratory	458	(59)	227	(66)	231	(53)	<0.001*	144	(56)	314	(60)	0.183*
Urinary	60	(8)	20	(6)	40	(9)		15	(6)	45	(9)	
Intra-abdominal	147	(19)	40	(12)	107	(24)		56	(22)	91	(17)	
Other	113	(14)	54	(16)	59	(14)		42	(16)	71	(14)	
**Number of comorbidities, **n (%)												
0	369	(48)	150	(45)	219	(51)	0.212*	105	(41)	264	(51)	0.018*
1	207	(27)	93	(28)	114	(26)		73	(29)	134	(26)	
>1	193	(25)	93	(28)	100	(23)		77	(30)	116	(23)	
**ICU length of stay**, med (IQR)	9	(5-16)	8	(5-15)	10	(5-17)	0.483^£^	6	(3-14)	10	(7-17)	**<0.001**^£^
**28-day mortality, **n (%)	257	(33)	66	(19)	191	(44)	<0.001*					

Differences in the patient profile according to the severity of sepsis (severe sepsis and septic shock) and 28 days outcome (death or alive) are shown.

IQR: interquartile range; SAPS: Simplified Acute Physiological Score; SD: standard deviation.

# t-test; * Pearson chi-square test; £ Mann-Whitney Test.

Compliance with the six-hour bundle actions was: (1) 62% for serum lactate measured; (2) 69% for fluids administration; (3) 77% for microbiology specimen collection prior to administration of antibiotics; (4) 48% for blood cultures collection; (5) 52% for antibiotics administration; (6) 78% for vasopressors administration; (7) 56% for CVP measurement; (8) 17% for ScvO2 measurement; (9) 52% for dobutamine administration. Only 12% (94 out of 778) of the patients with severe sepsis completed actions 1 to 6 ('core bundle')

Collecting blood cultures was the only action associated with a significant decrease in the 28-days mortality [OR = 0.57, 95% confidence interval (CI) = 0.38 to 0.84]. When adjusted for severity of sepsis, SAPS II score, number of comorbidities, type of hospital and type of ICU, the adjusted ORs for collecting blood cultures and giving vasopressors were significantly protective (Table 3).

**Table 3:** Odds ratio for 28-days mortality for each action of the bundle completed in patients with severe sepsis

	Odds ratio SIMPLE (95% CI)	*P *value	Odds ratio ADJUSTED (95% CI)	*P *value
**Individual actions of the core bundle**				
1) Serum lactate measured in the first six-hours	0.84 (0.61; 1.15)	0.272	0.74 (0.51; 1.06)	0.102
2) Fluids administered to achieve a MAP >65 mmHg	1.03 (0.73; 1.44)	0.870	0.95 (0.64; 1.41)	0.812
3) Specimens collected for microbiology before antibiotic therapy	0.80 (0.55; 1.15)	0.228	0.71 (0.47; 1.07)	0.101
4) Blood cultures done	**0.57 (0.38; 0.84)**	**0.005**	**0.53 (0.34; 0.83)**	**0.005**
5) Antibiotic therapy administered in the first hour after diagnosis	0.91 (0.67; 1.25)	0.561	0.85 (0.59; 1.21)	0.355
6) Vasopressors administered to achieve a MAP >65 mmHg	0.67 (0.42; 1.06)	0.089	**0.51 (0.30; 0.88)**	**0.014**
**Actions, 1 to 6, completed**				
0 to 2	1.00		1.00	
3 to 5	0.86 (0.62; 1.18)	0.601	0.73 (0.51; 1.05)	0.091
Fully completed	0.61 (0.36; 1.02)	0.061	**0.44** **(0.24; 0.80)**	**0.006**

The odds ratio is presented for each action of the bundle as single and grouped and it is adjusted for severity of sepsis, SAPS II, number of comorbidities, type of hospital and type of ICU for patients with severe sepsis. These results are based on 778 patients with severe sepsis and septic shock on ICU admission. CI: confidence interval; MAP: mean arterial pressure; SAPS: Simplified Acute Physiological Score.

For the overall severe septic patients, the full completion of the first six actions of the bundle was associated with a significant decrease in the 28-day mortality (adjusted OR = 0.44; 95% CI = 0.24 to 0.80). This corresponds to six patients needed to be treated to save one life.

In the subgroup of septic shock patients, completing the first six actions was also associated with a significant decrease in the 28-days mortality (adjusted OR = 0.49; 95% CI = 0.25 to 0.95; Table 4) as well as partial completion of the bundle (3 to 5 actions), although not reaching statistical significance (adjusted OR = 0.73; 95% CI = 0.51 to 1.05). Different categorisation of the groups of bundle completion did not alter the results.

**Table 4:** Odds ratio for 28-days mortality for each action of the bundle completed in patients with septic shock

	Odds ratio SIMPLE (95% CI)	*P *value	Odds ratio ADJUSTED (95% CI)	*P *value
**Individual actions of the bundle**				
1) Serum lactate measured in the first 6-hours	0.68 (0.46; 1.02)	0.064	0.64 (0.40; 1.03)	0.064
2) Fluids administered to achieve a MAP >65 mmHg	0.82 (0.52; 1.28)	0.383	1.01 (0.60; 1.70)	0.984
3) Specimens collected for microbiology before antibiotic started	**0.61 (0.37; 0.98)**	**0.041**	**0.57 **(**0.33**; **0.97**)	**0.037**
4) Blood cultures done	0.52 (0.32; 0.84)	0.008	**0.50 **(**0.29**; **0.88**)	**0.016**
5) Antibiotic therapy administered in the first hour after diagnosis	0.83 (0.56; 1.23)	0.356	0.77 (0.49; 1.21)	0.258
6) Vasopressores administered to achieve a MAP >65 mmHg	0.54 (0.30; 0.97)	0.038	**0.52 **(**0.28**; **0.99**)	**0.048**
7) Was CVP measured?	**0.62 (0.41; 0.94)**	**0.023**	0.74 (0.47; 1.18)	0.207
8) SvcO2 measured?	0.85 (0.50; 1.46)	0.853	0.76 (0.40; 1.44)	0.396
9) Inotropes administered	0.98 (0.56; 1.70)	0.931	0.94 (0.49; 1.80)	0.848
**Bundle**				
Bundle completed with actions 1) through 6) versus partial or non completed	**0.51** **(0.29; 0.90)**	**0.021**	**0.49** **(0.25; 0.95)**	**0.036**
Bundle completed with the 9 actions versus partial or non completed	0.51 (0.21; 1.26)	0.146	0.41 (0.14; 1.19)	0.101

The odds ratio is presented for each variable as single and grouped and it is adjusted for SAPS II, number of comorbidities, type of hospital and type of ICU for patients with septic shock. These results are based on 437 patients with septic shock. CI: confidence interval; CVP: central venous pressure; MAP: mean arterial pressure; SAPS: Simplified Acute Physiological Score; SvcO2: central venous oxygen saturation.

The overall 28-days mortality among severe septic patients was 33%. This unadjusted mortality rate increase to 34% in the group of patients that did not complete all the first six interventions of the six-hour bundle ('core bundle') and decreased to 25% in those who did but this difference was not statistically significant (*P *= 0.099)

Patients with septic shock had an increasing number of actions completed (Table 5), and shorter time interval to perform them, particularly blood cultures drawn, antibiotics administered and ICU admission The median time from hospital admission to ICU admission was the same in the group of survivals and non-survivals (13 hours, *P *= 0.876).

**Table 5:** Comparison between the group of patients that completed actions 1 to 6 and the groups that did not

	Total n = 778		0-2 Completed n = 288		3-5 Completed n = 396		Fully completed n = 94		*P *value
**Age**, mean (SD)	61	(17)	61	(17)	61	(17)	62	(16)	*0.836*^#^
**SAPS II**, mean (SD)	51	(19)	51	(19)	51	(18)	52	(19)	*0.770*^#^
**Gender, **n (%)									
Female	280	(36)	102	(35)	148	(37)	30	(32)	0.592*
Male	498	(64)	186	(65)	248	(63)	64	(68)	
**Number of comorbidities, **n (%)									
0	369	(48)	153	(53)	175	(45)	41	(44)	0.180*
1	207	(27)	74	(26)	104	(27)	29	(30)	
>1	193	(25)	61	(21)	108	(28)	24	(26)	
**Severity of sepsis**, n (%)									
Severe sepsis	341	(44)	154	(53)	156	(39)	31	(33)	<0.001*
Septic shock	437	(56)	134	(47)	240	(61)	63	(67)	
**Type of hospital**, n (%)									
Community	289	(37)	132	(46)	126	(32)	31	(37)	0.001*
University	489	(63)	156	(54)	270	(68)	63	(67)	
**Type of ICU**, n (%)									
Medical	231	(30)	97	(34)	117	(30)	17	(18)	***0.016***
Mixed	547	(70)	191	(66)	279	(70)	77	(82)	
**ICU length of stay, **median (IQR)	9	(5-16)	9	(5-15)	9	(5-16)	9	(6-17)	*0.579*^£^
**Hospital admission-blood cultures (hours), **median (IQR)	7	(2-23)	21	(9-40)	6	(2-18)	2	(1-3)	***<0.001***^£^
**Hospital admission-antibiotics administration (hours), **median (IQR)	5	(2-13)	9	(4-19)	5	(2-11)	3	(2-4)	***<0.001***^£^
**Hospital-ICU admission (hours), **median (IQR)	13	(4-38)	27	(2-54)	10	(3-30)	4	(2-11)	***<0.001***^£^
**28-day outcome**, n (%)									
Dead	257	(33)	104	(36)	129	(33)	24	(26)	0.064*
Alive	521	(67)	184	(64)	267	(67)	70	(74)	

IQR: interquartile range; SAPS: Simplified Acute Physiological Score; SD: standard deviation.

# One-way analysis of variance test; *Pearson chi-square test; £ Kruskal Wallis test.

## Discussion

### Main findings

The full completion with interventions 1 to 6 - core bundle - was associated with a significant decrease in the odds of 28-days mortality (adjusted OR = 0.44, *P *= 0.006).

We did not find a significant benefit of partial bundle completion, although there was a tendency towards it. But the main goal should really be to complete the whole bundle gaining the synergy of the bundle elements performed in unison rather than each one independently.

Only 12% of our patients fully completed the core bundle, but this study started immediately after the publication of SSC recommendations and the bundles definition [4,5]. Other studies have reported initial low compliance following the publication of international guidelines, such as the management of ST elevation acute myocardial infarction or the management of stroke [19,20].

In fact, a recent study on the impact of a national educational program on the process of care for severe septic patients [21] showed an initial improvement in compliance with SSC guidelines that dropped to the initial low compliance rates one year after the intervention. However, time for simple interventions such as serum lactate measurement, collection of blood cultures and administration of antibiotics remained shorter, suggesting that the easier the process the higher the penetration in clinical practice - therefore the core bundle should be the very first approach on-site to the severe septic patients.

The core bundle includes: stratification of sepsis through serum lactate measurement, specimen collection (including blood cultures) for microbiology followed by broad-spectrum antibiotic administration and fluids and vasopressors administration as needed to obtain a MAP over 65 mmHg, simple actions that should be performed immediately, and should prompt expert help in septic shock.

Our low compliance with the bundle could also indicate that doctors may not have been aware of the severity of the sepsis at the time of presentation. In fact, patients that had the core bundle completed in the first six hours were, in some way, more successful in attracting earlier medical attention, as both the time to specific interventions, such as blood cultures and antibiotics, and the time to ICU admission were significantly shorter among them. This was most likely due to the fact that these patients were more severely ill (Table 5) and therefore more prone to receive medical attention earlier. The routine measurement of serum lactate (as an early marker of tissue hypoperfusion) at the initial clinical assessment of patients with suspected infection could identify those with cardiovascular dysfunction at an early phase before overt clinical septic shock develops and at a time when therapeutic interventions (six-hour bundle) would be more effective.

The observed reduction in mortality was similar to that described in other studies, which also introduced modifications to the currently recommended SSC six-hour bundle [21-23], reinforcing the need to adapt SSC recommendations to the local settings, gaining a significant beneficial effect on severe sepsis mortality.

### Number needed to treat

The NNT is defined as the number of patients who must be treated to prevent one patient from experiencing the adverse effects of the disease being studied [24]. The magnitude of the NNT, six patients, in our study is similar to what has been found in other studies that compare mortality in septic patients before and after the implementation of SSC bundles. Otero and colleagues [25] review the impact of implementing early goal-directed therapy in severe sepsis, in 12 centres, incorporating a total of 1298 patients and reaching a NNT of 5. The 12 centres enrolled between 38 and 330 patients, and studied different parameters of the SSC bundles: early goal-directed therapy (with invasive monitoring of CVP and ScvO2) included control of focus of infection (particularly early antibiotic administration) and even 24-hour SSC bundle components (such as tight glucose control and steroids in septic shock), making a comparison between them difficult. In this review, the NNT varied between 3 (implementation of early goal-directed therapy [26]) and 11 patients (implementation of a multidisciplinary sepsis team, that besides early goal-directed therapy provide control of focus of infection and glucose control [27]).

### Strengths

This is a multi-centre study on sepsis involving 41% of all available Portuguese ICU beds that enrolled nearly 900 patients. These patients were quite homogeneous regarding the primary diagnosis - only CAS patients were considered. It was performed over a one-year period eliminating any bias related to seasonal variation.

Time 0 was clearly defined as the hospital arrival time, eliminating the influence of individual physician's assessment [22,23,28], making data more objective and comparable between units. However, selecting hospital arrival time instead of 'sepsis recognition time' may have biased the results towards lower compliance.

In fact, time 0 has been the subject of great debate [29]. Some authors [22] consider time 0 as the moment when the patient becomes hypotensive or when serum lactate is 4 mmol/L or higher, while others consider time 0 as the moment of the diagnosis, regardless of how long the patient has been in hospital [23]. The use of such different definitions may markedly affect the assessment of compliance to interventions, making comparison between studies difficult. Moreover, and of more concern, definitions considering time of diagnosis (of sepsis, of hypotension, of high lactates level) as the starting point may give doctors a false reassurance. Therefore, time 0 should be assumed as an operational criterion in the patients' interest and, for community-acquired infection, should preferably be defined as the time of hospital admission.

### Limitations

Our study included only patients that were admitted to the ICU with CAS, and it could be that some patients in the emergency department received early adequate antibiotics and correct fluid resuscitation and got a good response and were therefore not included in the study, creating a bias against the treatment. On the other hand some patients could be compliant with the bundle just because their severity was low and they did not need fluids or vasoactive drugs to achieve the resuscitation endpoint, despite the adjustments this could be a bias favoring the treatment.

Our patients were not randomised and so there is always the possibility of unforeseen and unmeasured biases that could affect the results. However, there are ethical limitations to perform randomised trials examining the utility of some of the bundle actions. Therefore, data from well-conducted prospective studies are best suited to assess this question. Also, a number of key variables such as severity of disease and patient previous health status were taken into consideration and used for adjustment during the statistical analysis. What would be possible are cluster-randomised studies examining the effects of various approaches and intensity in implementing the guidelines in comparable settings.

We did not control for differences in medical knowledge about the management of severe sepsis patients. The participation in this study was performed on a voluntary basis, so the doctors involved are probably the ones with more interest in the field and more willing to be updated.

Invasive monitoring, dependent on the placement of a central venous catheter (CVC) in the first six-hours of hospital admission, had a very low compliance among the septic shock group. Therefore, it is not surprising that the effect we found (adjusted OR = 0.41, *P *= 0.101) was not statistically significant. The low compliance with monitoring of CVP and ScvO2 is probably dependent on the need of specialised help for placing the central venous line. In emergency departments where the patients are seen mainly by undifferentiated doctors the clinical pathway for severe sepsis should include calling for expert help immediately, if serum lactate is high and/or if blood pressure remains low after the first fluid bolus, to initiate aggressive hemodynamic resuscitation.

## Conclusions

Early sepsis recognition (eg. serum lactate measurement), optimisation of oxygen delivery (eg. fluid resuscitation and vasopressors) and infection treatment (eg. appropriate antibiotics and infection control, preceded by blood cultures) may result in a significant reduction in 28-day mortality. Due to the potential preventive effect of the core bundle completion on patient mortality, locally driven organisational interventions are urgently needed, along with a wide educational campaign to change behaviours - addressed to doctors and nurses working in the emergency department, ICU and general medical and surgical wards - if mortality is to be improved. The implementation of the severe sepsis clinical pathway should include calling for expert help for every patient with cardiovascular dysfunction to assure aggressive hemodynamic resuscitation. Similarly, to the golden-hour concept for trauma [30], acute myocardial infarction [31] and stroke [32], we propose the sepsis six-hour 'golden bundle' concept for early diagnosis and intervention as it may be a golden approach to reduce mortality.

## Key messages

• Prevalence of CAS among ICU admissions was 24%.

• In the first six hours of hospital admission compliance with serum lactate measurement, fluids administration, specimens collection for microbiology before antibiotic administration, blood cultures collection, antibiotics administration within the first hour of the diagnosis and vasopressors administration when needed, were associated with an OR for 28-days mortality of 0.44 (95% CI = 0.24 to 0.80) in severe sepsis patients.

• The magnitude of the NNT - six patients to save one life - of these simple actions should prompt locally driven organisational interventions if mortality is to be improved. The implementation of a severe sepsis clinical pathway should include calling for expert help for every patient with cardiovascular dysfunction to assure aggressive hemodynamic resuscitation.

## Abbreviations

CAS: community-acquired sepsis; CI: confidence interval; CVP: central venous pressure; NNT: number needed to treat; OR: odds ratio; SACiUCI: community-acquired sepsis admitted to ICU; SAPS II: simplified acute physiological score; ScvO2: central venous oxygen saturation; SOFA: sequential organ failure assessment; SSC: surviving sepsis campaign.

## Competing interests

The authors declare that they have no competing interests.

## Authors' contributions

All authors have made substantial contribution on the conception, design and acquisition of data and/or analysis and interpretation of data, as well as in the drafting, revising and final approval of the version to be published.
